# A Clinical Teaching Blended Learning Program to Enhance Registered Nurse Preceptors’ Teaching Competencies: Pretest and Posttest Study

**DOI:** 10.2196/18604

**Published:** 2020-04-24

**Authors:** Xi Vivien Wu, Yuchen Chi, Umadevi Panneer Selvam, M Kamala Devi, Wenru Wang, Yah Shih Chan, Fong Chi Wee, Shengdong Zhao, Vibhor Sehgal, Neo Kim Emily Ang

**Affiliations:** 1 Alice Lee Centre for Nursing Studies Yong Loo Lin School of Medicine National University of Singapore Singapore Singapore; 2 Department of Education and Practice, Nursing Service Tan Tock Seng Hospital Singapore Singapore; 3 School of Computing National University of Singapore Singapore Singapore; 4 University of California Berkeley Berkeley, CA United States

**Keywords:** blended learning, case-based learning, clinical pedagogy, clinical teaching competency, web-based program, nurse preceptor

## Abstract

**Background:**

Clinical nursing education provides opportunities for students to learn in multiple patient care settings, receive appropriate guidance, and foster the development of clinical competence and professionalism. Nurse preceptors guide students to integrate theory into practice, teach clinical skills, assess clinical competencies, and enhance problem-solving and critical thinking skills. Previous research has indicated that the teaching competencies of nurse preceptors can be transferred to students’ clinical learning to enhance their clinical competencies.

**Objective:**

This study aimed to develop a clinical teaching blended learning (CTBL) program with the aid of web-based clinical pedagogy (WCP) and case-based learning for nurse preceptors and to examine the effectiveness of the CTBL program on nurse preceptors’ clinical teaching competencies, self-efficacies, attitudes toward web-based learning, and blended learning outcomes.

**Methods:**

A quasi-experimental single-group pretest and posttest design was adopted. A total of 150 nurse preceptors participated in the CTBL program, which was conducted from September 2019 to December 2019. A set of questionnaires, including the clinical teaching competence inventory, preceptor self-efficacy questionnaire, attitudes toward web-based continuing learning survey, and e-learning experience questionnaire, was used to assess the outcomes before and after the CTBL program.

**Results:**

Compared with the baseline, the participants had significantly higher total mean scores and subdomain scores for clinical teaching competence (mean 129.95, SD 16.38; *P*<.001), self-efficacy (mean 70.40, SD 9.35; *P*<.001), attitudes toward web-based continuing learning (mean 84.68, SD 14.76; *P*<.001), and blended learning outcomes (mean 122.13, SD 14.86; *P*<.001) after the CTBL program.

**Conclusions:**

The CTBL program provides a comprehensive coverage of clinical teaching pedagogy and assessment strategies. The combination of the WCP and case-based approach provides a variety of learning modes to fit into the diverse learning needs of the preceptors. The CTBL program allows the preceptors to receive direct feedback from the facilitators during face-to-face sessions. Preceptors also gave feedback that the web-based workload is manageable. This study provides evidence that the CTBL program increases the clinical teaching competencies and self-efficacies of the preceptors and promotes positive attitudes toward web-based learning and better blended learning outcomes. The health care organization can consider the integration of flexible learning and intellect platforms for preceptorship education.

## Introduction

### Background

Clinical nursing education provides opportunities for students to learn in multiple patient care settings, receive appropriate guidance, and foster the development of clinical competence and professionalism. In a preceptorship program, a nursing student is assigned to a registered nurse preceptor who is responsible for clinical guidance. Preceptors nurture the development of clinical knowledge, skills, and professional attitudes in nursing students through guiding, role modeling, and facilitating professional development [1]. The preceptors’ levels of competence impact students’ learning experiences and clinical competencies. However, preceptors often lack teaching knowledge and experience, which leads to role ambiguity and unfamiliarity with clinical education systems [2]. Hence, training in clinical teaching pedagogy is recommended to assist with preceptors’ professional development. Innovative continuing education promotes the professional development of health care professionals and fits into their busy working schedules [3]. Technology alone is not able to meet the diverse learning needs of health care professionals in the clinical environment. Nevertheless, blended learning is recognized as an effective approach to provide an alternative learning strategy and promote the integration of theory and practice [4]. It is paramount that continuing education courses integrate technology, increase the flexibility and responsiveness of the workforce, and offer alternative means to attend the courses [5].

### Literature Review

#### Registered Nurse Preceptors’ Teaching Competencies and Self-Efficacies

The transition from a nursing student to a professional nurse is challenging and may lead to anxiety and frustration, which consequently result in job dissatisfaction, low productivity, and attrition [6]. It is critical to retain nurses at the workplace to address the global nursing shortage. Nurse preceptors play important roles in clinical education. Preceptors facilitate the clinical learning of students through the process of socialization by helping students to adjust to their working cultures and environment and become functional members of their teams. Positive preceptorship experience prepares nursing students for smooth transitions into the reality of practice [7]. In addition, preceptors help nursing students to acquire a sense of professional identity through caring and teaching [8]. What students learn from nurse preceptors will be reflected in their daily practices when they become independent nurses [8]. Hence, the competency levels of nurse preceptors directly affect the quality of future nurses.

Previous studies indicate that it is crucial to prepare preceptors to be pedagogically ready to embark on the preceptorship process [2,9-11]. Researchers have reported that preceptors who have greater knowledge of preceptor roles tend to display higher self-efficacies and confidence in their capabilities of clinical teaching [12]. Furthermore, the way nursing students are precepted shapes workplace cultures and environments, which may help with the issue of retaining nurses at the workplace to address the global nursing shortage [11,13]. Therefore, nurse preceptors’ teaching competency is positively related to the quality of future nurses, which translates to improved patient safety, quality of care, and staff retention [14,15].

#### Web-Based Learning for Health Care Professionals

Nowadays, it is crucial for health care workers to access continuous learning opportunities to update their knowledge and skills. However, health care institutions always face challenges in supporting their staff for continuous professional developments, such as costs of training, staff absence from clinical areas, and limited time to attend courses [16]. Electronic learning (e-learning) using information technology as a medium provides an alternative to build virtual platforms for learners’ interactions [17]. With the advancement of information technology, e-learning platforms are able to demonstrate outcomes comparable with those of face-to-face programs by using interactive simulation videos and discussion forums [18-21]. E-learning is flexible and allows nurses to learn at their own pace [22]. Thus, it is evident as an effective alternative means to train health care professionals within resource-limited settings [23].

Although e-learning has been widely used in hospital training programs, there are still emerging concerns. It requires a certain level of computer literacy and skills. Nurses who have not taken web-based courses require more support to adapt to e-learning platforms [22,24,25]. Some studies highlighted concerns regarding the limitations of learners’ assessments and feedback when using e-learning programs, which can significantly impact learning outcomes [26,27]. In addition, learners may feel isolated and disengaged during e-learning, which may result in a poor completion rate [28].

#### Blended Learning and a Case-Based Approach for Health Care Professionals

Blended learning involves a systematic combination of face-to-face interactions and technology-mediated interactions among learners, facilitators, and resources [29]. Compared with e-learning sessions, the teaching and learning styles of face-to-face sessions should be redesigned to fit into the learning needs of health care professionals [30]. Blended learning can engage case studies, group discussions, and debates to emphasize social elements during the session [16,30]. This will increase human interactions and peer support to motivate learners in the learning process [28,31].

During face-to-face sessions, case-based learning is a highly advocated learning approach in health professional education because it encourages learners to apply knowledge to solve problems [32,33]. It allows learners to either discuss in groups or to have a discussion guided by a facilitator after a case presentation [34]. Case-based learning promotes active and reflective learning and facilitates learners to improve their critical thinking and problem-solving skills [34,35]. Blended learning brings e-learning and face-to-face learning together and provides learners with exposure to various learning experiences. Studies have highlighted the advantages of blended learning, including more flexibility, less time restrictions, greater pedagogic richness, and more cost-effectiveness [36,37]. It can support diverse learners to meet their learning needs. In addition, blended learning helps learners improve their sense of autonomy and responsibility and allows facilitators to maintain learners’ engagement and motivation [16,38,39].

A review of the literature shows that the number of blended learning studies has been limited, and the area of study focuses more on learners’ and facilitators’ satisfaction, perception, and experience [16,38,40]. As an innovative approach, blended learning has not been validated for nurse preceptor training. Hence, this study aimed to develop a clinical teaching blended learning (CTBL) program with the aid of web-based clinical pedagogy (WCP) and case-based learning for nurse preceptors and to examine the effectiveness of the CTBL program on nurse preceptors’ clinical teaching competencies, self-efficacies, attitudes toward web-based learning, and blended learning outcomes.

## Methods

### Research Questions

The study was designed to address the following research questions:

1. What is the effect of the CTBL program on the clinical teaching competencies of nurse preceptors?
2. What is the effect of the CTBL program on nurse preceptors’ self-efficacies in conducting clinical teaching and assessment?
3. What is the effect of the CTBL program on nurse preceptors’ attitudes toward web-based continuing learning?
4. What are the effects of the CTBL program on nurse preceptors’ blended learning outcomes?

### Study Design and Setting

A single-group prospective pretest and posttest design was adopted for this study. The CTBL program with the aid of WCP and case-based learning for nurse preceptors was developed. The study was conducted at an acute tertiary hospital located in the central region of Singapore.

### Participants

The target population was nurses who were assigned the roles of clinical teaching and assessments of the nursing students. The inclusion criteria included nurse preceptors who (1) were guiding nursing students for clinical teaching and assessments, (2) were aged 21 years and older, (3) had not completed the clinical teaching course provided by the hospital, and (4) used smart mobile devices (smartphones, tablets, or laptops) in their daily lives. The exclusion criteria included nurse preceptors who did not use smart mobile devices. A total of 150 registered nurse preceptors were eligible for participation in this study.

The sample size needs to be determined by the number of participants who are required to maintain statistical power for statistical tests used in data analysis. The sample size was calculated using a paired *t* test, with an adjusted alpha of .0167 (Bonferroni approach) and an estimated effect size and power of 0.8. With the use of the IBM SPSS Version 25 statistical tool G*power [41], the sample size was calculated to be 120. Factoring at a 20.0% attrition rate, the estimated sample size was 144. A total of 150 participants were recruited for this study.

### Intervention

**Figure 1:**
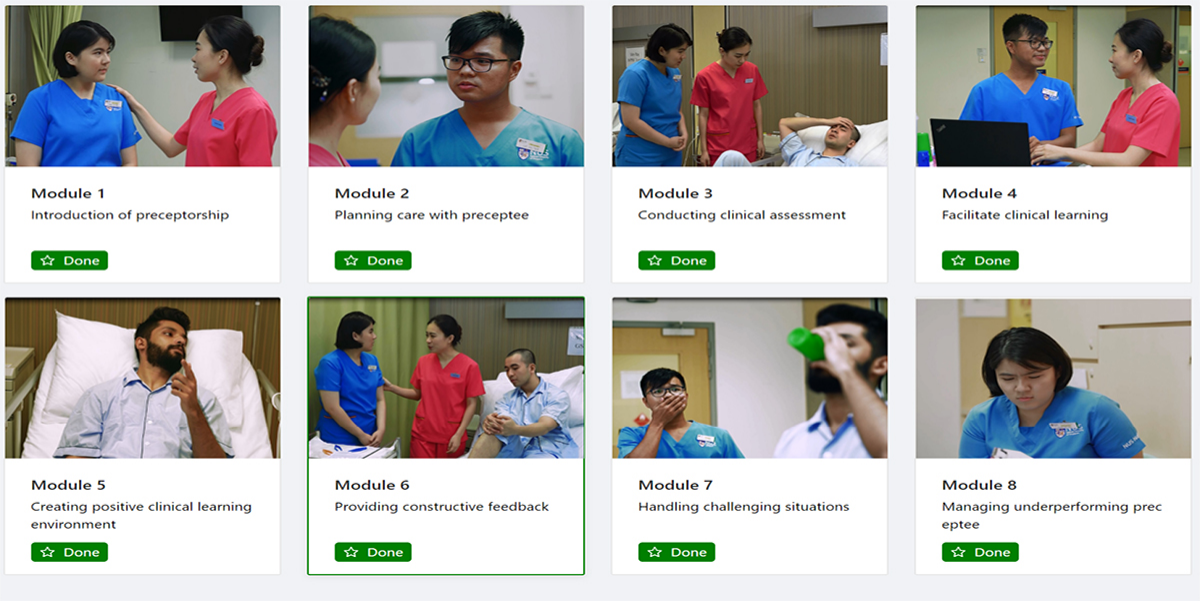
Modules in the web-based clinical pedagogy program.

The CTBL program consists of a WCP program and a face-to-face case-based learning workshop. The CTBL program was carried out as a 1-day workshop (8 hours). In the morning session, the participants were given access and time to attend the WCP program. The WCP program was developed by a research team using a 3-step process: integrate the theoretical framework, evidence from systematic review, and feedback from content validity test by experts and the result of the pilot test with the nurse preceptors. The WCP program consists of 8 modules: (1) introduction of preceptorship, (2) planning care with preceptee, (3) conducting clinical assessment, (4) facilitating clinical learning, (5) creating a positive clinical learning environment, (6) providing constructive feedback, (7) handling challenging situations, and (8) managing underperforming preceptees. The details of the WCP program have been reported in another paper published by the team [21] (Figure 1).

In the afternoon session, face-to-face case-based learning was conducted for preceptors by a facilitator who was also a nurse educator. In total, 3 case studies were used in case-based learning. The team developed case studies based on common situations that preceptors might encounter in the process of clinical teaching. Preceptors were divided into small groups to discuss, present, and share how they planned to manage such situations. The group discussion and presentation were facilitated by the nurse educator. Learner-to-learner and learner-to-facilitator communication and interaction were maintained throughout the case discussion. Table 1 presents a sample of the case study. Case-based learning is frequently used in medical and nursing education. Studies have demonstrated that case-based learning promotes group processes, collaborative learning, critical thinking, and decision making. In addition, learners are encouraged to participate actively in the discussion and critique of each other’s work [34,42].

**Table 1:** Sample of the case study.

Case study	Case description	Guiding questions
Case study 1	You are the preceptor of a final-year nursing student. After completing the hospital orientation program, your nurse manager introduces your preceptee to you. After a discussion with your preceptee, you find out that she was previously trained at a different hospital and had never done any attachment in ABC hospital.	Share how you will welcome your preceptee Discuss some concerns that you may have about your preceptee How would you assess her level of competency for her expected skills?
Case study 2	You have a preceptee who is a matured learner. You realize that he had 20 years of working experience in a financial sector as a manager. However, he was retrenched from the previous job and took up nursing as a midcareer switch job. You experience disagreements between you and your preceptee frequently. At the same time, you realize that he asks valid questions at work in an unacceptable tone. You have other colleagues giving feedback to you that your preceptee is not a good team player.	Discuss the possible reasons for the preceptee’s behavior Share how you plan to work with your preceptee
Case study 3	You are the preceptor to a nursing student from a foreign country. She has just completed the hospital orientation program. You have observed that she appeared withdrawn and lost in the ward when caring for patients. She demonstrates an unenthusiastic attitude toward learning and you have never seen her ask questions.	Discuss possible reasons for her behavior Explore her learning needs Discuss your plan in approaching the situation

### Outcome Measures

Sociodemographic profiles, including gender, age, race, educational level, job title, clinical department, area of specialization, years of working experience as a registered nurse, and years of experience in clinical teaching, were obtained using structured questionnaires. In total, 4 instruments were used to evaluate the learning outcomes of the nurse preceptors. The face and content validity for the clinical teaching competence inventory (CTCI), preceptor self-efficacy questionnaire (PSEQ), attitudes toward a web-based continuing learning survey (AWCLS), and e-Learning experience questionnaire (LEQ) were assessed by the research team and the experts committee in this study.

#### Clinical Teaching Competence Inventory

The CTCI was developed in Taiwan [43]. It consists of 4 domains of teaching and assessment competencies and 31 items. The 4 domains are student evaluation (assessment), goal setting and individual teaching, teaching strategies, and demonstration of organized knowledge. The CTCI was psychometrically tested, and the results indicated that the instrument has adequate content validity (scale content validity index =0.75) and internal consistency of reliability (Cronbach alpha=.88) for assessing clinical teaching and assessment behavior in practice settings.

#### Preceptor Self-Efficacy Questionnaire

The PSEQ [44] consists of 21 items and uses a 4-point scale (Cronbach alpha=.93). The participants used the PSEQ to rate their confidence in teaching strategies, learning critical thinking, challenging situations, providing feedback and evaluations, and overall confidence in precepting nursing students.

#### Attitudes Toward a Web-Based Continuing Learning Survey

The AWCLS was developed based on relevant studies and the technology acceptance model [45]. There are 4 dimensions and 18 items using a 7-point Likert scale in the survey. The 4 dimensions are (1) the perceived usefulness domain assesses the nurses’ perceptions of the extent to which they perceive that the influence of web-based continuing learning is useful, (2) the perceived ease of use domain evaluates the nurses’ perceptions of the extent to which web-based continuing learning is easy to use, (3) the behavior domain measures the nurses’ willingness to take up web-based continuing learning, and (4) the affection domain assesses the nurses’ perceptions regarding positive feelings about web-based continuing learning. The results of the alpha coefficients (Cronbach alpha=.96) for the scales indicated that the AWCLS is appropriate for evaluating nurses’ attitudes toward web-based continuing learning [46].

#### e-Learning Experience Questionnaire

The LEQ is used to measure the outcomes of a blended learning environment that combines e-learning and face-to-face interactions [47]. The instrument consists of 32 items that are grouped into 9 domains: quality of teaching in an e-learning context, participants’ interaction and engagement, clarity of goals and standards for online component, quality of online resources, appropriateness of the assessment in an e-learning context, appropriateness of workload related to online materials and activities, issues related to participants management, degree to which online materials and activities support face-to-face learning, and overall satisfaction with the quality of online materials and activities. The Cronbach alpha values range from .61 to .84 for different segments [47].

### Ethical Considerations

The National Health Group Domain Specific Review Board (approval number: 2018/00138) approved the ethical aspect of the study. Permission and support to conduct the study were obtained from the senior management of the hospital. A participant information sheet was given to each potential participant with details on the aims and procedures of the study. The participants were reassured that participation in the study was voluntary and that their identities would remain anonymous. They were informed that they had the right to withdraw from the study at any time.

### Data Collection Procedure

Recruitment emails were sent to each potential participant. The research team explained the research process and obtained consent from potential participants. Nurse preceptors were invited to complete the sociodemographic profile and questionnaires (CTCI, PSAI, AWCLS, and LEQ) before they started the CTBL program (baseline). Posttest questionnaires were administered at the end of the CTBL program. A total of 6 training workshops were conducted between September 2019 and December 2019. Each workshop consisted of 20 to 30 participants. Data were collected using *MySurvey*, a secured web-based platform subscribed by the university. *MySurvey* operated on Verint Enterprise Feedback Management 15.2, and the website was deployed on a campus on-premise server and maintained by the information technology center of the university.

### Data Analysis

Data were retrieved from the *MySurvey* platform. A total of 2 research assistants checked the accuracy of the data. SPSS 25.0 (SPSS institute) was used to analyze the data. Descriptive statistics, such as frequency, mean, and SD, were used to analyze the sociodemographic data and study outcomes. The paired *t* test was used to compare the pre- and postintervention score changes at a significance level of .05. The final analysis was based on 150 participants who had completed all the data collection. The normality of outcome variables was examined using the Shapiro-Wilk test and quantile-quantile plot normal distribution graphics and by visually inspecting the histogram generated. Normality was also determined through skewness values divided by SE and kurtosis divided by SE. Normality was indicated by a value of less than −2.0 or more than 2.0 [48].

## Results

**Figure 2:**
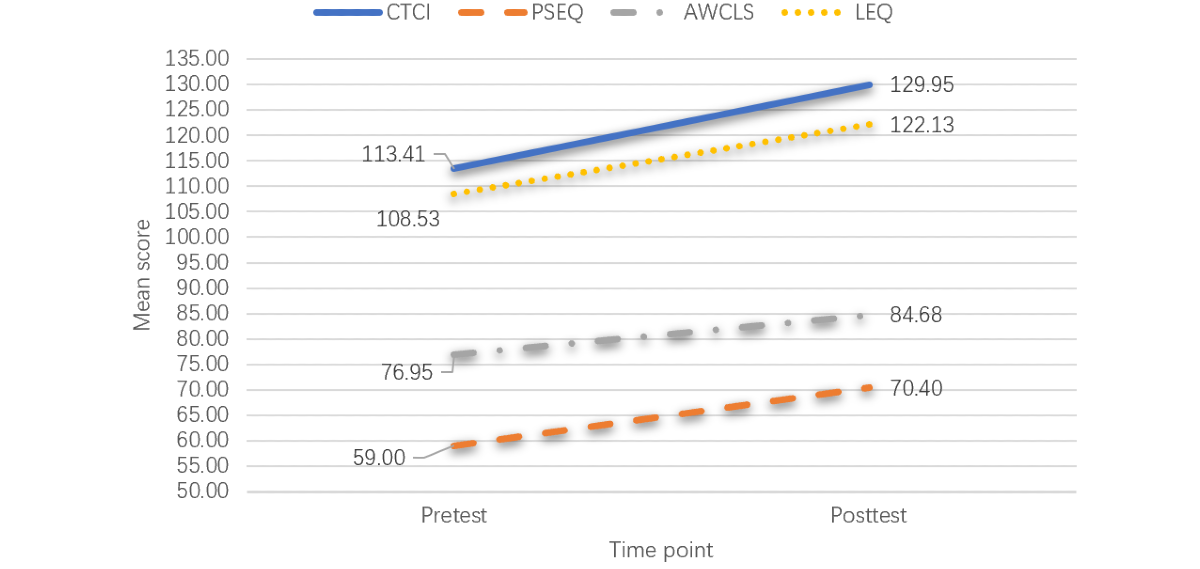
Mean score pretest and posttest (N=150) for AWCLS. AWCLS: attitudes toward a web-based continuing learning survey; CTCI: clinical teaching competence inventory; LEQ: e-Learning experience questionnaire; PSEQ: preceptor self-efficacy questionnaire.

The study examined the impact of the CTBL program on nurse preceptors’ clinical teaching competencies, self-efficacies, attitudes toward web-based learning, and blended learning outcomes. The results demonstrated significant improvement in most of the outcome measures, as illustrated in Figure 2.

### Sociodemographic Characteristics

Table 2 demonstrates the sociodemographic characteristics of the participants. More than 90.0% (140/150) of the participants were female, and the mean age was 31.6 years. The ethnicity distributions were Chinese (38/150, 25.3%), Malay (26/150, 17.3%), Indian (29/150, 19.3%), Filipino (52/150, 34.7%), and other ethnic groups (5/150, 3.3%). The majority of the participants (108/150, 72.0%) were graduates who had a Bachelor of Nursing degree, and 28.0% (42/150) of the participants were diploma or certificate holders. More than two-thirds of the participants (103/150, 68.7%) were senior/registered nurses, and one-third of them (47/150, 31.3%) were enrolled nurses. The average working experience was 8 years. The majority of the participants had relevant clinical teaching experiences, ranging from 1 to 3 years (98/150, 65.3%) to 3 to 10 years (32/150, 21.3%). However, 12.7% (19/150) of the participants did not have any clinical teaching experience.

**Table 2:** Sociodemographic characteristics of the participants (N=150).

Characteristic			Value
Age (years), mean (SD)			31.6 (6.4)
**Gender, n (%)**			
	Female	140 (93.3)	
	Male	10 (6.7)	
**Ethnicity, n (%)**			
	Chinese	38 (25.4)	
	Malay	26 (17.3)	
	Indian	29 (19.3)	
	Filipino	52 (34.7)	
	Others	5 (3.3)	
**Education level, n (%)**			
	Bachelor of nursing	108 (72.0)	
	Diploma of nursing	31 (20.7)	
	Certificate of nursing	11 (7.3)	
Years of working, mean (SD)			8 (5.0)
**Clinical teaching experience (years), n (%)**			
	0	19 (12.7)	
	1-3	98 (65.3)	
	3-6	20 (13.3)	
	7-10	12 (8.0)	
	>10	1 (0.7)	
**Current position, n (%)**			
	Enrolled nurse	47 (31.3)	
	Registered nurse	68 (45.4)	
	Senior registered nurse	35 (23.3)	

### Clinical Teaching Competencies

The participants had significant increases in their clinical teaching competency scores after completion of the CTBL program (mean 129.95, SD 16.38; *P*<.001) compared with the baseline (mean 113.41, SD 16.67; *P*<.001). Overall, the postintervention scores were significantly higher than the preintervention scores in all 4 subdomains: student evaluation (mean 37.57, SD 4.80; *P*<.001), goal setting and individual teaching (mean 30.18, SD 4.26; *P*<.001), teaching strategies (mean 38.22, SD 5.084; *P*<.001), and demonstration of organized knowledge (mean 16.63, SD 2.26, *P*<.001; Table 3).

**Table 3:** Mean scores for the clinical teaching competence inventory, preceptor self-efficacy questionnaire, and attitudes toward a web-based continuing learning survey, before and after the intervention (N=150).

Measure		Preintervention, mean (SD)	Postintervention, mean (SD)	Difference within group, mean (95% CI)	*t* test (*df*=149)	*P* value
**Clinical teaching competency inventory**						**<.001**
	Student evaluation	31.98 (5.56)	37.57 (4.80)	5.59 (4.63-6.54)	11.53	
	Goal setting and individual teaching	25.74 (5.18)	30.18 (4.26)	4.44 (3.58-5.30)	10.2	
	Teaching strategies	34.79 (5.26)	38.22 (5.084)	3.43 (2.39-4.46)	6.53	
	Demonstration of organized knowledge	14.45 (2.45)	16.63 (2.26)	2.19 (1.73-2.64)	9.54	
	Total score	113.41 (16.67)	129.95 (16.38)	16.53 (13.37-19.70)	10.33	
**Preceptor self-efficacy questionnaire**						**<.001**
	Total score	59.00 (11.05)	70.40 (9.35)	10.56 (8.70-12.42)	11.23	
**Attitude toward web-based continuing learning survey**						**<.001**
	Perceived usefulness	25.74 (5.30)	28.25 (5.42)	2.51 (1.70-3.33)	6.07	
	Perceived ease of use	20.79 (4.11)	22.85 (3.85)	2.05 (1.41-2.70)	6.27	
	Behavior	14.96 (3.43)	16.73 (3.24)	1.77 (1.24-2.31)	6.53	
	Affection	15.45 (3.70)	16.85 (3.47)	1.39 (0.85-1.94)	5.04	
	Total score	76.95 (15.33)	84.68 (14.76)	7.73 (5.49-9.98)	6.81	

### Preceptor Self-Efficacies

The mean score changes of the preceptors’ self-efficacies from pre- to postintervention are shown in Table 3. The participants had a significantly higher total score for self-efficacy after the CTBL program (mean 70.40, SD 9.35; *P*<.001) than at the baseline (mean 59.00, SD 11.05; *P*<.001).

### Attitudes Toward Web-Based Continuing Learning

The mean changes in attitudes toward web-based continuing learning are shown in Table 3. The total mean score for attitudes toward web-based continuing learning increased significantly postintervention (mean 84.68, SD 14.76; *P*<.001) compared with the baseline (mean 76.95, SD 15.33; *P*<.001). The postintervention scores were significantly higher than baseline in all 4 subdomains: perceived usefulness (mean 28.25, SD 5.42; *P*<.001), perceived ease of use (mean 22.85, SD 3.85; *P*<.001), behavior (mean 16.73, SD 3.24; *P*<.001), and affection (mean 16.85, SD 3.47; *P*<.001) toward web-based continuing learning.

### Blended Learning Outcomes

Multimedia Appendix 1 indicates the mean score changes of the blended learning outcomes from pre- to postintervention. The instrument is a Likert scale that uses *strongly disagree*, *disagree*, *neutral*, *agree*, and *strongly agree*. To facilitate the display, *strongly disagree* and *disagree* were combined as *disagree* and *agree* and *strongly agree* were combined as agree. The participants had a significantly higher total mean score for blended learning outcomes after the CTBL program (mean 122.13, SD 14.86; *P*<.001) than at the baseline (mean 108.53, SD 14.07; *P*<.001). In general, the postintervention scores were significantly higher than the preintervention scores, in most of the subdomains:

- Subdomain 1: quality of teaching in e-learning context (mean 25.62, SD 2.80; *P*<.001),
- Subdomain 2: participants’ interaction and engagement (mean 14.93, SD 2.24; *P*<.001),
- Subdomain 3: clarity of goals and standards for online component (mean 11.78, SD 1.63; *P*<.001),
- Subdomain 4: quality of online resources (mean 15.84, SD 2.19; *P*<.001),
- Subdomain 5: appropriateness of assessment in e-learning context (mean 10.85, SD 1.49; *P*<.001),
- Subdomain 7: issues related to participants management (mean 11.95, SD 1.60; *P*<.001),
- Subdomain 8: degree to which online materials and activities support face-to-face learning (mean 15.95, SD 2.13; *P*<.001), and
- Subdomain 9: overall satisfaction with the quality of online materials and activities (mean 4.09, SD 0.63; *P*<.001).

However, for subdomain 6: appropriateness of workload related to online materials and activities, although slightly improved, there was no significant difference in the score before and after the intervention (mean 9.49, SD 1.80; *P*=.16).

## Discussion

### Principal Findings

The results of the study have provided empirical evidence for our hypotheses in that nurse preceptors who received the CTBL program scored higher in terms of clinical teaching competencies, self-efficacies in conducting clinical teaching and assessment, attitudes toward web-based learning, and blended learning outcomes. In a systematic review, it was identified that the younger generation was more likely to access web-based programs [49]. In this study, the mean age of the participants was 31.6 years. This is considered a relatively young group of nurses. Hence, they could be more positive and acceptable to new modes of learning, such as blended and web-based platforms.

Our study suggested that the blended learning approach provided pedagogical benefits to the participants to enhance their competencies and self-efficacies in clinical teaching. The importance of having a competent clinical preceptor is universally recognized, as it forms the basis of a good clinical mentorship to enhance the learning outcomes of a student in clinical practice [50]. Self-efficacy is important because job satisfaction and confidence not only impact preceptors but also impact the retention, turnover, and job satisfaction of newly recruited nurses. Good clinical teaching and mentorship often result in positive outcomes, such as increased job satisfaction, productivity, quality of care, and patient safety for students or newly recruited nurses [51,52]. Most preceptors perceived improvement of professional knowledge and teaching competencies as the most important benefit of preceptorship and motivation for precepting students [53,54]. However, in a study by Seo et al [55], the majority of the preceptors viewed monetary compensation as a valuable incentive, whereas the others preferred support for education and promotion in career. Hence, appropriate extrinsic compensation, for example, opportunities for promotion and monetary rewards could be considered to attract and encourage nurses to take on the role of preceptors.

The blended learning approach has shown evidence of its relevance and usefulness in medical and nursing training [56,57]. It has been shown to be an effective method of training in health care professionals with high levels of learner satisfaction [58-60]. On the basis of Tobin’s [61] 3-dimensional framework to evaluate participants’ perception of the web-based environment, we evaluated the CTBL program. First, the *emancipatory activities* dimension of the CTBL program allowed the participants to control the pace, place, and depth of learning by offering the WCP program, and the participants could have more flexibility in learning and reviewing the contents of the program. Second, the *coparticipatory activities* dimension (the case-based approach), provides a platform for the participants to explore and discuss various options in clinical teaching and assessment among the peer learners and with the instructors. Third, in the *qualia* dimension of the CTBL program, the participants enjoyed the program in general, and this is reflected in the overall improvement in competency and self-efficacy in clinical teaching and positive attitudes toward web-based learning and blended learning outcomes.

The CTBL program consists of WCP and case-based learning. This program replaced traditional lectures in the classroom using a web-based asynchronous learning mode. Research shows that health care professionals appreciate asynchronous, digital teaching and learning [62]. In fact, a challenge for educators is to meet the learning needs of 21st century learners in providing more flexible digital learning platforms [63]. However, case-based learning allows participants to interact with peers and instructors through face-to-face discussions. In fact, the results of the LEQ indicated that the participants valued this active learning part of the CTBL program. Research has shown that the higher the learner’s engagement level, the greater the learners’ learning potential [64]. In fact, a number of preceptors were actively engaged in discussions during the development process of the CTBL program. The early involvement of the preceptors contributed to the identification of the learning needs and preferences of the targeted participants, the verification of the contents of the program, and creative teaching pedagogies. Researchers have identified five important elements in the process of developing new technological learning methods: clarifications of learners’ expectations, help to recognize the bigger picture, stimulation of interactions, creations of a structure, and context-specific contents [65].

In the LEQ, items 1, 3, 11, 15, and 22 were phrased negatively. Hence, these items were reverse coded in the data analysis. There were 3 items in the LEQ that did not demonstrate significant changes after the intervention. For item 1, “to do well in the online quizzes all you really need is a good memory” (*P*=.18), 40 participants (40/150, 26.7%) selected *agree* postintervention compared with 20 participants (20/150, 13.3%) at the baseline. In fact, good performance in quizzes does not require only good memory but should also reflect learners’ mastery of the contents. The quizzes in the CTBL program were designed in such a way that learners should apply knowledge learned from the module to solve problems in the scenarios. Research has demonstrated that less experienced preceptors are more unwilling to fail incompetent students in clinical settings because of a lack of confidence in their own capabilities [66]. Junior preceptors often feel impeded in their decision-making abilities because of lack of clinical teaching knowledge and experiences. The scenarios in the WCP and case-based approach provided a platform for the nurse preceptors to have a real sense of clinical situations and how they could provide guidance and support to their preceptee and develop their judgment skills. Quizzes after each scenario further evaluated the preceptors’ ability to apply learnt knowledge to solve issues that they may encounter in real clinical settings.

For item 11, “the workload for the online component of this unit of study is too heavy” (*P*=.47), the number of participants who selected *disagree* increased from 12.0% (18/150) at baseline to 37.3% (56/150) postintervention. After the CTBL program, 3 times the number of participants were inclined to reasonable workload for web-based learning. This could be an indicator that learners need to have real hands-on experience to change their preperceived idea of web-based learning. The workload of web-based learning is a debatable hot topic. Faculties argued that web-based education appears to have acquired an unstoppable momentum [67]. Notably, limited studies have explored the workload of web-based learning.

Items 3 and 15 are related to the feedback provided to the learners. For item 3, “I received too much feedback online from my teacher/facilitator” (*P*=.02), although there was a significant change, it is interesting to note that 34.6% (52/150) of participants selected *disagree* postintervention compared with 22.0% (33/150) of participants at baseline. More participants concurred that the feedback from the facilitator was reasonable. For item 15, “I didn’t receive enough helpful online feedback from my teacher/facilitator” (*P*=.29), 28.0% (42/150) of participants selected *disagree* postintervention compared with 14.0% (21/150) of participants at baseline. Similarly, the number of participants who experienced good quality feedback doubled. This shows that the CTBL program provided both sufficient and good quality feedback to the learners. It is evident that efficient interactions between the facilitator and learners contribute to the creation of a positive learning climate [68]. Learners value timely, quality feedback and frequent interaction in the blended learning environment.

### Limitations

The study population was restricted to one health care institution in Singapore. Therefore, it may limit the generalizability of the study. However, as the participants were nurse preceptors from various clinical settings, such as medical, surgical, orthopedic, cardiac, and renal, this could be a good representation of nurses from a wide range of clinical settings. One of the limitations of the study is the lack of a control group. Hence, practitioners and management should exercise caution when applying the results of this study. It would be beneficial to use randomized controlled trials in a variety of health care settings. Thus, stronger evidence on the effectiveness of the CTBL program can be generated.

### Implication for Practice

The CTBL program is a further enhancement of the WCP program [21], which provides comprehensive coverage on clinical teaching pedagogy and assessment strategies. In addition, case-based learning provides a platform for preceptors to interact and discuss clinical teaching experiences with their peers and facilitators. The combination of WCP and the face-to-face approach provides a variety of learning modes that fit into the diverse learning needs of the preceptors. The CTBL program allows preceptors to receive direct feedback from facilitators during face-to-face sessions. This study also sheds some light on web-based workload as the preceptors gave feedback that the web-based workload was manageable. Future research can explore the workload of web-based learning and effective ways of providing feedback on the web because these areas are understudied.

For course developers, it is important to consider learning styles, learning needs, approaches, and possible platforms in the initial design phase, and logistic issues, such as Wi-Fi connection, servers, and physical classrooms, should be taken into consideration. In addition, the comprehensive approach of the evaluation process enabled the provision of scientific evidence for the effectiveness of the program. More research can explore the cost-effectiveness of the program in terms of saving manpower, cost, time, academic standards, etc.

The CTBL program can be used in other acute hospitals, community hospitals, primary care settings, and higher education institutions to prepare nurse preceptors to be pedagogically ready. Nurse preceptors need resources and support to develop their teaching-coaching capabilities. Comprehensive clinical teaching preparation programs and a variety of learning platforms, such as web-based and face-to-face discussions, provide open communication lines and viable resources for active preceptors. Ongoing support should be offered to preceptors, such as support from nurse managers and recognition and compensatory systems, to encourage and reward them to take up preceptorship roles. Such resources promote preceptors in embracing clinical teaching pedagogy and the spirit of life-long learning. Hence, nursing students have more enriched clinical learning experiences through guidance from preceptors with competent clinical teaching skills.

### Conclusions

Currently, with an increasing reliance of the clinical nursing education system on preceptors to facilitate students’ learning, it is imperative to provide preceptors with essential pedagogical knowledge. Clinical teaching preparation will enhance role satisfaction and sustain the willingness to perform as a preceptor. The CTBL program is an innovative clinical teaching program for nurse preceptors. The program consists of a WCP and a face-to-face case-based learning workshop, which meet the diverse learning needs of the preceptors and provide a flexible platform that fits into the busy working schedules of preceptors. This study provides empirical evidence that the CTBL program increases the clinical teaching competencies and self-efficacies of preceptors and promotes positive attitudes toward web-based learning and better blended learning outcomes. Health care organizations can consider the integration of flexible learning and intellectual platforms for preceptorship education, such as web-based, case-based, and blended learning approaches.

## Appendix

Multimedia Appendix 1 Mean scores for the e-Learning experience questionnaire before and after the intervention (N=150).

## Abbreviations

AWCLS: attitudes toward a web-based continuing learning survey
CTBL: clinical teaching blended learning
CTCI: clinical teaching competence inventory
e-learning: electronic learning
LEQ: e-Learning experience questionnaire
PSEQ: preceptor self-efficacy questionnaire
WCP: web-based clinical pedagogy
