# Supplementary material for: A Clinical Teaching Blended Learning Program to Enhance Registered Nurse Preceptors’ Teaching Competencies: Pretest and Posttest Study
Source: J Med Internet Res. 2020 Apr 24;22(4):e18604. doi: 10.2196/18604 (PMC7210493; doi:10.2196/18604)
Supplement: Multimedia Appendix 1 [file jmir_v22i4e18604_app1.docx]

**Multimedia Appendix Table 1.** Mean scores for the e-Learning experience questionnaire before and after the intervention (N=150).

| Subdomain/item | | Preintervention | | | | Postintervention | | | | *t* test value | *P* value |
| --- | --- | --- | --- | --- | --- | --- | --- | --- | --- | --- | --- |
|  | | Mean (SD) | Likert scale response, n (%) | | | Mean (SD) | Likert scale response, n (%) | | |  |  |
|  | |  | Disagree | Neutral | Agree |  | Disagree | Neutral | Agree |  |  |
|  | |  |  |  |  |  |  |  |  |  |  |
| **Quality of teaching in e-learning^a^ context** | | | | | | | | | | | |
|  | Subdomain total | 23.33 (2.33) | —^c^ | — | — | 25.62 (2.80) | — | — | — | 8.80_149_ | <.001 |
|  | 3(R)^b^ | 3.05 (0.76) | 33 (22.0) | 81 (54.0) | 36 (24.0) | 2.83 (0.94) | 52 (34.6) | 67 (44.7) | 31 (20.7) | −2.34_149_ | .02 |
|  | 4 | 3.41 (0.73) | 11 (7.3) | 67 (44.7) | 72 (48.0) | 3.90 (0.69) | 2 (1.3) | 38 (25.3) | 110 (73.4) | 6.55_149_ | <.001 |
|  | 5 | 3.47 (0.70) | 9 (6.0) | 62 (41.3) | 79 (52.7) | 4.01 (0.70) | 3 (2.0) | 26 (17.3) | 111 (22.7) | 7.03_149_ | <.001 |
|  | 9 | 3.45 (0.71) | 5 (3.3) | 77 (51.3) | 68 (45.4) | 4.02 (0.64) | 0 (0.0) | 29 (19.3) | 121 (80.7) | 7.30_149_ | <.001 |
|  | 12 | 3.38 (0.59) | 5 (3.3) | 83 (55.3) | 62 (41.4) | 3.87 (0.70) | 2 (1.3) | 42 (28.0) | 106 (70.7) | 7.15_149_ | <.001 |
|  | 15(R) | 3.15 (0.68) | 21 (14.0) | 87 (58.0) | 42 (28.0) | 3.05 (1.04) | 42 (48.0) | 60 (40.0) | 48 (32.0) | −1.07_149_ | .29 |
|  | 28 | 3.43 (0.78) | 9 (6.0) | 71 (47.3) | 70 (46.7) | 3.93 (0.63) | 3 (2.0) | 26 (17.3) | 121 (80.7) | 6.66_149_ | <.001 |
| **Participants interaction and engagement** | | | | | | | | | | | |
|  | Subdomain total | 13.23 (1.91) | — | — | — | 14.93 (2.24) | — | — | — | 7.72_149_ | <.001 |
|  | 7 | 3.37 (0.73) | 10 (6.7) | 75 (49.3) | 66 (44.0) | 3.84 (0.72) | 3 (2.0) | 43 (28.7) | 104 (69.3) | 6.18_149_ | <.001 |
|  | 16 | 3.09 (0.61) | 17 (11.3) | 103 (68.7) | 30 (20.0) | 3.42 (0.83) | 12 (8.0) | 72 (48.0) | 66 (44.0) | 4.06_149_ | <.001 |
|  | 18 | 3.38 (0.71) | 8 (5.4) | 77 (51.3) | 65 (43.4) | 3.85 (0.70) | 3 (2.0) | 40 (26.7) | 107 (71.3) | 6.65_149_ | <.001 |
|  | 21 | 3.39 (0.65) | 7 (4.6) | 79 (52.7) | 64 (42.7) | 3.81 (0.69) | 3 (2.0) | 43 (28.7) | 104 (69.3) | 5.86_149_ | <.001 |
| **Clarity of goals and standards for online component** | | | | | | | | | | | |
|  | Subdomain total | 10.38 (1.69) | — | — | — | 11.78 (1.63) | — | — | — | 8.29_149_ | <.001 |
|  | 13 | 3.43 (0.64) | 7 (4.7) | 73 (48.6) | 70 (46.6) | 3.93 (0.64) | 1 (0.7) | 33 (22.0) | 116 (77.3) | 7.32_149_ | <.001 |
|  | 19 | 3.47 (0.68) | 5 (3.3) | 74 (49.3) | 71 (47.4) | 3.91 (0.66) | 2 (1.3) | 34 (22.7) | 114 (76.0) | 6.39_149_ | <.001 |
|  | 29 | 3.47 (0.71) | 6 (4.0) | 74 (49.3) | 70 (46.7) | 3.95 (0.64) | 1 (0.7) | 32 (21.3) | 117 (78.0) | 6.53_149_ | <.001 |
| **Quality of online resources** | | | | | | | | | | | |
|  | Subdomain total | 13.89 (2.30) | — | — | — | 15.84 (2.19) | — | — | — | 9.20_149_ | <.001 |
|  | 8 | 3.49 (0.68) | 4 (2.7) | 74 (49.3) | 72 (48.0) | 3.99 (0.67) | 2 (1.3) | 28 (18.7) | 120 (80.0) | 7.05_149_ | <.001 |
|  | 17 | 3.41 (0.76) | 11 (7.4) | 68 (45.3) | 71 (47.3) | 3.85 (0.67) | 1 (0.7) | 41 (27.3) | 108 (72.0) | 5.99_149_ | <.001 |
|  | 20 | 3.48 (0.72) | 9 (6.0) | 65 (43.3) | 76 (50.7) | 3.98 (0.64) | 1 (0.7) | 29 (19.3) | 120 (80.0) | 7.65_149_ | <.001 |
|  | 23 | 3.50 (0.72) | 5 (3.3) | 68 (45.3) | 77 (51.4) | 4.03 | 0 (0.0) | 25 (16.7) | 125 (83.3) | 7.74_149_ | <.001 |
| **Appropriateness of assessment in e-learning context** | | | | | | | | | | | |
|  | Subdomain total | 9.53 (1.17) | — | — | — | 10.85 (1.49) | — | — | — | 10.09_149_ | <.001 |
|  | 1(R) | 2.65 (0.90) | 70 (46.7) | 60 (40.0) | 20 (13.3) | 2.78 (1.14) | 68 (45.3) | 42 (28.0) | 40 (26.7) | 1.37_149_ | .18 |
|  | 10 | 3.45 (0.72) | 8 (5.3) | 70 (46.7) | 72 (48.0) | 4.07 (0.65) | 0 (0.0) | 26 (17.3) | 124 (82.7) | 8.89_149_ | <.001 |
|  | 26 | 3.43 (0.66) | 6 (4.0) | 75 (50.0) | 69 (46.0) | 4.00 (0.60) | 0 (0.0) | 27 (18.0) | 123 (82.0) | 8.61_149_ | <.001 |
| **Appropriateness of workload related to online materials and activities** | | | | | | | | | | | |
|  | Subdomain total | 9.25 (0.98) | — | — | — | 9.49 (1.80) | — | — | — | 1.41_149_ | .16 |
|  | 11(R) | 3.03 (0.57) | 18 (12.0) | 108 (72.0) | 24 (16.0) | 2.95 (1.15) | 56 (37.3) | 49 (32.7) | 45 (30.0) | −0.73_149_ | .47 |
|  | 14 | 3.43 (0.69) | 10 (6.7) | 66 (44.0) | 74 (49.3) | 3.96 (0.66) | 1 (0.7) | 33 (22.0) | 116 (77.3) | 7.27_149_ | <.001 |
|  | 22(R) | 2.79 (0.62) | 42 (28.0) | 99 (66.0) | 9 (6.0) | 2.57 (0.97) | 72 (48.7) | 56 (37.3) | 21 (14.0) | −2.43_149_ | .02 |
| **Issues related to participants management** | | | | | | | | | | | |
|  | Subdomain total | 10.89 (1.71) | — | — | — | 11.95 (1.60) | — | — | — | 6.65_149_ | <.001 |
|  | 2 | 3.66 (0.68) | 5 (3.3) | 48 (32.0) | 97 (64.7) | 3.97 (0.63) | 28 (17.6) | 97 (64.7) | 25 (16.7) | 4.84_149_ | <.001 |
|  | 6 | 3.69 (0.78) | 6 (4.0) | 49 (32.7) | 95 (63.3) | 4.01 (0.64) | 1 (0.7) | 27 (18.0) | 122 (81.3) | 4.06_149_ | <.001 |
|  | 31 | 3.55 (0.77) | 9 (6.0) | 54 (36.0) | 87 (58.0) | 3.97 (0.61) | 1 (0.7) | 27 (18.0) | 122 (81.3) | 6.23_149_ | <.001 |
| **Degree to which online materials and activities support face-to-face learning** | | | | | | | | | | | |
|  | Subdomain total | 13.84 (2.24) | — | — | — | 15.95 (2.13) | — | — | — | 9.71_149_ | <.001 |
|  | 24 | 3.51 (0.72) | 6 (4.0) | 67 (44.7) | 77 (51.3) | 3.91  (0.61) | 4 (2.7) | 29 (19.3) | 117 (78.0) | 5.69_149_ | <.001 |
|  | 25 | 3.47 (0.66) | 5 (3.3) | 73 (48.7) | 72 (48.0) | 4.03 (0.60) | 0 (0.0) | 27 (18.0) | 123 (82.0) | 8.02_149_ | <.001 |
|  | 27 | 3.36 (0.74) | 11 (7.3) | 77 (51.3) | 62 (41.3) | 3.99 (0.61) | 0 (0.0) | 29 (19.3) | 121 (80.7) | 8.81_149_ | <.001 |
|  | 30 | 3.51 (0.74) | 7 (4.7) | 69 (46.0) | 74 (49.3) | 4.03 (0.58) | 0 (0.0) | 23 (15.3) | 127 (74.7) | 7.50_149_ | <.001 |
| **Overall satisfaction with the quality of online materials and activities** | | | | | | | | | | | |
|  | Subdomain total | 3.51 (0.69) | — | — | — | 4.09 (0.63) | — | — | — | 8.93_149_ | <.001 |
|  | 32 | 3.51 (0.69) | 5 (3.3) | 70 (46.7) | 75 (50.0) | 4.09 (0.63) | 1 (0.7) | 20 (13.3) | 129 (86.0) | 8.93_149_ | <.001 |
| Total score | | 108.53 (14.07) | — | — | — | 122.13 (14.86) | — | — | — | 9.49_149_ | <.001 |

^a^e-learning: electronic learning.

^b^R indicates that the item is reversely coded.

^c^— indicates that the value is not applicable
